# To fast, or not to fast before chemotherapy, that is the question

**DOI:** 10.1186/s12885-018-4245-5

**Published:** 2018-03-27

**Authors:** Riccardo Caccialanza, Emanuele Cereda, Francesco De Lorenzo, Gabriella Farina, Paolo Pedrazzoli, on behalf of the AIOM-SINPE-FAVO Working Group

**Affiliations:** 1 Clinical Nutrition and Dietetics Unit, Fondazione IRCCS Policlinico San Matteo, Viale Golgi 19, 27100 Pavia, Italy; 2 Italian Federation of Volunteer-based Cancer Organizations, Rome, Italy; 3 Department of Oncology, ASST Fatebenefratelli Sacco, Milan, Italy; 4 Medical Oncology Unit, Fondazione IRCCS Policlinico San Matteo, Pavia, Italy

**Keywords:** Fasting, Chemotherapy, Calorie restriction, Malnutrition, Sarcopenia

## Abstract

Fasting in disease prevention and treatment has recently become a popular topic, particularly in the context of oncology. Unfortunately, the growing attention paid by the media has created a background of speculations and ambiguous messages.

The attitude towards the role of fasting in cancer patients should be very cautious, as the risk of malnutrition/sarcopenia and disinformation may be associated with this approach.

Whether the results obtained by fasting in the cellular and animal models can be transferred to cancer patients is still to be ascertained. At the moment, more preclinical studies are required to determine in which cancers, at which stage, and in what combinations fasting, fasting-mimicking diets or caloric restriction mimetics may prove effective.

So, despite the “rumors” of marketing and media, nowadays fasting and calorie restriction around CT represent only a promising intuition, which requires proper efforts and time to be validated by evidence-based clinical data.

## Background

Fasting for disease prevention and during treatment has recently become a popular topic, particularly in the context of oncology [1]. Unfortunately, the growing attention paid by the media has created a background of speculation and ambiguous messages.

Fasting before chemotherapy (CT) was shown to protect healthy cells from treatment toxicity by reducing the expression of some oncogenes, such as RAS and the AKT signaling pathway [2]. This reduction is mediated by the decrease of circulating insulin-like growth factor 1 (IGF-1) and glucose. In addition, starvation and calorie restriction activate other oncogenes in cancer cells, induce autophagy, and decrease cellular growth rates while increasing sensitivity to antimitotic drugs [2].

In particular, the molecular mechanisms involved in autophagy following fasting and calorie restriction, represent potentially novel ways of designing more effective anti-cancer treatment strategies [3]. However, autophagy’s role in cancer is complicated by the fact that, although tumor-suppressive in healthy cells, it may promote malignancy in cancer cells [3].

Immunological mechanisms, too, are altered by starvation. In particular, caloric restriction increases the activity of CD-8 cytotoxic lymphocytes and inhibits T-regulatory cell function, leading to increased autophagy and cell death [4].

Calorie restriction is also able to modulate the tumor microenvironment, by allowing enhanced drug delivery, by reducing substrate availability for cancer cells, and by reducing circulating growth factors and inflammation [5].

## Main text

Most of the available studies regarding fasting or calorie restriction in cancer are still pre-clinical, having been conducted in vitro or in animals. These studies have shown that starvation enhances p53’s effects on proapoptotic gene expression and apoptosis in breast cancer and melanoma cells [6].

Fasting was shown to potentiate the efficacy of tyrosine kinase inhibitors by inhibiting the mitogen-activated protein kinase signaling pathway [7], and to synergize with Sorafenib in hampering hepatocellular carcinoma cell growth and glucose uptake, while normalizing the expression levels of genes commonly altered by Sorafenib itself [8].

A study tested the effects of starvation on glioma cells and in mice with subcutaneous or intracranial models of glioma. Fasting for 48 h induced cancer sensitization to radiotherapy or CT in cells and rats and led to extended survival in starved animals [9]. Similar results were found in pancreatic cancer cells and in xenografted mice subjected to 24 h starvation prior to gemcitabine injection [10], although progression of certain tumors was not prevented following short-term calorie or macronutrient restriction [11].

On the other hand, it seems that fasting could be replaced by the administration of drugs called “caloric restriction mimetics”, such as hydroxycitrate and spermidine, which without causing weight loss, improve the efficacy of CT by inducing autophagy by depleting regulatory T cells from the tumor bed [12].

A group of researchers recently started to develop the so called “fasting mimicking diets”, to attain fasting-like effects while providing micronutrients to minimize the burden of fasting [13]. These dietary approaches were developed both in humans and rodents. In humans, the program consists of a plant-based 5-day diet. On day 1, almost 1000 kcal are provided, while almost 700 kcal are allowed on days 2 to 5 independently of patients’ age, body weight and composition [13], which makes this approach medically questionable.

It should be emphasized that the evidence provided by human studies is still very limited.

The feasibility of fasting for 24, 48 and 72 h before and during CT, was tested in 20 cancer patients [14]. Fasting-related toxicities were limited, and a trend indicating reduced DNA damage in leucocytes from subjects fasting for at least 48 h, was detected. A small pilot randomized study evaluated the effects of fasting in 13 CT patients with HER2-negative breast cancer [15]. Hematological toxicity and DNA damage in peripheral blood mononuclear cells were lower in the fasting group, while no differences in non-hematological toxicities were detected.

A case series report with anecdotal value, showed that 10 patients with different cancer types undergoing CT did not experience side effects caused by fasting itself other than hunger and dizziness [16]. Moreover, 6 of these patients reported reduced fatigue and gastrointestinal side effects while fasting. Finally, a recent randomized study assessed the effects of 3 cycles of fasting-mimicking diet in 100 healthy subjects, and showed that common risk factors for cardiovascular diseases, diabetes, ageing and IGF-1 levels were reduced in the fasting group [17]. Regarding the latter, any association with reduced cancer risk cannot be inferred, as the exact predictive role of IGF-1 levels in different cancer types remains to be ascertained [18].

A very recent review underlined that several trials are currently underway to determine the potential for short-term fasting in reducing the side effects and enhancing the efficacy of CT, but revealed that, despite a considerable number of these having apparently been completed, the results have not yet been published [5].

We believe that the attitude towards the role of fasting in cancer patients should be very cautious, as two potential risks may be associated with this approach. The first of these is malnutrition and sarcopenia, which could be worsened by repeated or prolonged fasting episodes, and which are strongly associated with treatment-related toxicity, reduced response to cancer treatment, impaired quality of life and a worse overall prognosis in the most common cancer types [19, 20]. This is indeed a major concern, taking into account the high prevalence of malnutrition in cancer patients [21] and the lack of data on the impact of this treatment strategy in the presence of malnutrition, even from pre-clinical studies. Completed and ongoing human trials have only considered the exclusion of patients with low body mass index and weight loss [14–16], which overlooks the issue of sarcopenic obesity and its negative clinical implications [22]. Therefore, a clear indication of fasting’s real applicability, which could be limited to a very small set of patients, is not still available.

In light of this evidence, and considering the complete lack of clinical data, the most recent guide lines on nutritional support in cancer patients stated that fasting before or during CT is not recommended, particularly in malnourished patients or those at nutritional risk, not only because of the risk of malnutrition and sarcopenia, but also because patients might be tempted to prolong fasting episodes and chronically reduce calorie intake [23, 24]. This last aspect is strongly correlated with the second potential risk, which is disinformation.

Disinformation is a critical issue with regard to nutrition for cancer patients. Despite the lack of any evidence-based clinical evidence, hundreds of books and web sites promote anti-cancer diets and nutritional supplements to prevent or cure cancer. Also, fasting has apparently become a business, as fasting-mimicking diet kits have been recently commercialized on the web (http://l-nutra.com/) and many books regarding the clinical effects of starvation in cancer and other diseases are easily available and strongly promoted. This expanding market of “alternative” hypocaloric or fasting-mimicking diets with putative anti-cancer effects is a serious and potentially harmful problem, which may negatively interfere with cancer patients’ care, as these dietary regimens could decrease protein-calorie intake, leading to malnutrition and sarcopenia [23]. Moreover, the uncontrolled use of such unproven remedies could negatively interfere with active treatments.

Whether the results obtained by fasting in cellular and animal models are relevant to cancer patients remains to be ascertained. Furthermore, it will be vital to evaluate if the hypothesized benefits of fasting can positively impact the main clinical oncologic endpoints, like treatment response, toxicity, treatment tolerance, progression-free survival, overall survival and quality of life. At the moment, more preclinical studies are required to determine in which cancers, at which stage, and in what combinations these fasting, fasting-mimicking diets or caloric restriction mimetics may prove effective [5]. Future studies will have to take into consideration the risk of malnutrition and sarcopenia, the immunologic and metabolic state of the enrolled patients and may also focus on the potential of fasting in enhancing the response to lower doses of CT and radiotherapy. Another key issue which will require consideration is the need for establishing valid standard protocols that are able to correlate dietary approaches with chemotherapeutic treatments [25].

On the other hand, nutritional support has already been shown to improve quality of life and survival in advanced cancer patients [26], but there are still too few intervention trials on the efficacy of systematic nutritional support in patients receiving active anti-cancer treatments, especially during the early phases of disease, although with encouraging results [27–30]. Therefore, additional evidence on the “feeding approach” is also required.

## Conclusions

In conclusion, despite the “rumors” of marketing and media, the benefit of fasting and calorie restriction in CT patients, represents only a promising intuition, which requires proper effort and time to be validated by evidence-based clinical data. Indeed, the first step should be the identification of the right set of patients to whom this approach could be applied and who could really benefit from it. Meanwhile, we believe that priority should be given to guarantee to all cancer patients the right to receive comprehensive evidence-based clinical information on their nutritional status, together with prompt and appropriate nutritional counseling and/or support, apposite to their ensuing anticancer treatment while effectively treating or preventing malnutrition and sarcopenia [31].

## Abbreviations

AKT: Serine/threonine-specific protein kinase
CD8: Cluster of differentiation 8
CT: Chemotherapy
HER2: Human epidermal growth factor receptor 2
IGF-1: Insulin-like growth factor 1

## References

[1] Horne BD, Muhlestein JB, Anderson JL (2015). Health effects of intermittent fasting: hormesis or harm? A systematic review. Am J Clin Nutr.

[2] Longo VD, Mattson MP (2014). Fasting: molecular mechanisms and clinical applications. Cell Metab.

[3] Petibone DM, Majeed W, Casciano DA (2017). Autophagy function and its relationship to pathology, clinical applications, drug metabolism and toxicity. J Appl Toxicol.

[4] Englert JM, Powell JD (2016). Hunger pains: stimulating the appetite of the immune system for Cancer. Cancer Cell.

[5] O'Flanagan CH, Smith LA, McDonell SB, Hursting SD (2017). When less may be more: calorie restriction and response to cancer therapy. BMC Med.

[6] Shim HS, Wei M, Brandhorst S, Longo VD (2015). Starvation promotes REV1 SUMOylation and p53-dependent sensitization of melanoma and breast cancer cells. Cancer Res.

[7] Caffa I, D'Agostino V, Damonte P, Soncini D, Cea M, Monacelli F, Odetti P, Ballestrero A, Provenzani A, Longo VD, Nencioni A (2015). Fasting potentiates the anticancer activity of tyrosine kinase inhibitors by strengthening MAPK signaling inhibition. Oncotarget.

[8] Lo Re O, Panebianco C, Porto S, Cervi C, Rappa F, Di Biase S, Caraglia M, Pazienza V, Vinciguerra M (2018). Fasting inhibits hepatic stellate cells activation and potentiates anti-cancer activity of Sorafenib in hepatocellular cancer cells. J Cell Physiol.

[9] Safdie F, Brandhorst S, Wei M, Wang W, Lee C, Hwang S, Conti PS, Chen TC, Longo VD (2012). Fasting enhances the response of glioma to chemo- and radiotherapy. PLoS One.

[10] D'Aronzo M, Vinciguerra M, Mazza T, Panebianco C, Saracino C, Pereira SP, Graziano P, Pazienza V (2015). Fasting cycles potentiate the efficacy of gemcitabine treatment in in vitro and in vivo pancreatic cancer models. Oncotarget.

[11] Brandhorst S, Wei M, Hwang S, Morgan TE, Longo VD (2013). Short-term calorie and protein restriction provide partial protection from chemotoxicity but do not delay glioma progression. Exp Gerontol.

[12] Pietrocola F, Pol J, Vacchelli E, Rao S, Enot DP, Baracco EE, Levesque S, Castoldi F, Jacquelot N, Yamazaki T, Senovilla L, Marino G, Aranda F, Durand S, Sica V, Chery A, Lachkar S, Sigl V, Bloy N, Buque A, Falzoni S, Ryffel B, Apetoh L, Di Virgilio F, Madeo F, Maiuri MC, Zitvogel L, Levine B, Penninger JM, Kroemer G (2016). Caloric restriction mimetics enhance anticancer Immunosurveillance. Cancer Cell.

[13] Brandhorst S, Choi IY, Wei M, Cheng CW, Sedrakyan S, Navarrete G, Dubeau L, Yap LP, Park R, Vinciguerra M, Di Biase S, Mirzaei H, Mirisola MG, Childress P, Ji L, Groshen S, Penna F, Odetti P, Perin L, Conti PS, Ikeno Y, Kennedy BK, Cohen P, Morgan TE, Dorff TB, Longo VD (2015). A periodic diet that mimics fasting promotes multi-system regeneration, enhanced cognitive performance, and Healthspan. Cell Metab.

[14] Dorff TB, Groshen S, Garcia A, Shah M, Tsao-Wei D, Pham H, Cheng CW, Brandhorst S, Cohen P, Wei M, Longo V, Quinn DI (2016). Safety and feasibility of fasting in combination with platinum-based chemotherapy. BMC Cancer.

[15] de Groot S, Vreeswijk MP, Welters MJ, Gravesteijn G, Boei JJ, Jochems A, Houtsma D, Putter H, van der Hoeven JJ, Nortier JW, Pijl H, Kroep JR (2015). The effects of short-term fasting on tolerance to (neo) adjuvant chemotherapy in HER2-negative breast cancer patients: a randomized pilot study. BMC Cancer.

[16] Safdie FM, Dorff T, Quinn D, Fontana L, Wei M, Lee C, Cohen P, Longo VD (2009). Fasting and cancer treatment in humans: a case series report. Aging.

[17] Wei M, Brandhorst S, Shelehchi M, Mirzaei H, Cheng CW, Budniak J, Groshen S, Mack WJ, Guen E, Di Biase S, Cohen P, Morgan TE, Dorff T, Hong K, Michalsen A, Laviano A, Longo VD. Fasting-mimicking diet and markers/risk factors for aging, diabetes, cancer, and cardiovascular disease. Sci Transl Med. 2017;9(377) 10.1126/scitranslmed.aai8700 PMC6816332 28202779

[18] Anisimov VN, Bartke A (2013). The key role of growth hormone-insulin-IGF-1 signaling in aging and cancer. Crit Rev Oncol Hematol.

[19] Aapro M, Arends J, Bozzetti F, Fearon K, Grunberg SM, Herrstedt J, Hopkinson J, Jacquelin-Ravel N, Jatoi A, Kaasa S, Strasser F, ESMO (European School of Medical Oncology) (2014). Early recognition of malnutrition and cachexia in the cancer patient: a position paper of a European School of Oncology Task Force. Ann Oncol.

[20] Prado CM, Antoun S, Sawyer MB, Baracos VE (2011). Two faces of drug therapy in cancer: drug-related lean tissue loss and its adverse consequences to survival and toxicity. Curr Opin Clin Nutr Metab Care.

[21] Hébuterne X, Lemarié E, Michallet M (2014). Prevalence of malnutrition and current use of nutrition support in patients with cancer. JPEN.

[22] Carneiro IP, Mazurak VC, Prado CM (2016). Clinical implications of Sarcopenic obesity in Cancer. Curr Oncol Rep.

[23] Caccialanza R, Pedrazzoli P, Cereda E, Gavazzi C, Pinto C, Paccagnella A (2016). Nutritional support in cancer patients: a position paper from the Italian Society of Medical Oncology (AIOM) and the Italian Society of Artificial Nutrition and Metabolism (SINPE). J Cancer.

[24] Arends J, Bachmann P, Baracos V, Barthelemy N, Bertz H, Bozzetti F, Fearon K, Hütterer E, Isenring E, Kaasa S, Krznaric Z, Laird B, Larsson M, Laviano A, Mühlebach S, Muscaritoli M, Oldervoll L, Ravasco P, Solheim T, Strasser F, de van der Schueren M, Preiser JC (2017). ESPEN guidelines on nutrition in cancer patients. Clin Nutr.

[25] Cangemi A, Fanale D, Rinaldi G, Bazan V, Galvano A, Perez A, Barraco N, Massihnia D, Castiglia M, Vieni S, Bronte G, Mirisola M, Russo A (2016). Dietary restriction: could it be considered as speed bump on tumor progression road?. Tumour Biol.

[26] Cotogni P, De Carli L, Passera R, Amerio ML, Agnello E, Fadda M, Ossola M, Monge T, De Francesco A, Bozzetti F (2017). Longitudinal study of quality of life in advanced cancer patients on home parenteral nutrition. Cancer Med.

[27] Qiu M, Zhou YX, Jin Y, Wang ZX, Wei XL, Han HY, Ye WF, Zhou ZW, Zhang DS, Wang FH, Li YH, Yang DJ, Xu RH (2015). Nutrition support can bring survival benefit to high nutrition risk gastric cancer patients who received chemotherapy. Support Care Cancer.

[28] Gavazzi C, Colatruglio S, Valoriani F, Mazzaferro V, Sabbatini A, Biffi R, Mariani L, Miceli R (2016). Impact of home enteral nutrition in malnourished patients with upper gastrointestinal cancer: a multicentre randomised clinical trial. Eur J Cancer.

[29] Cereda E, Cappello S, Colombo S, Klersy C, Imarisio I, Turri A, Caraccia M, Borioli V, Monaco T, Benazzo M, Pedrazzoli P, Corbella F, Caccialanza R (2018). Nutritional counseling with or without systematic use of oral nutritional supplements in head and neck cancer patients undergoing radiotherapy. Radiother Oncol.

[30] Miyata H, Yano M, Yasuda T, Yamasaki M, Murakami K, Makino T, Nishiki K, Sugimura K, Motoori M, Shiraishi O, Mori M, Doki Y (2017). Randomized study of the clinical effects of ω-3 fatty acid-containing enteral nutrition support during neoadjuvant chemotherapy on chemotherapy-related toxicity in patients with esophageal cancer. Nutrition.

[31] Caccialanza R, De Lorenzo F, Gianotti L, Zagonel V, Gavazzi C, Farina G, Cotogni P, Cinieri S, Cereda E, Marchetti P, Nardi M, Iannelli E, Santangelo C, Traclò F, Pinto C, Pedrazzoli P (2017). Nutritional support for cancer patients: still a neglected right?. Support Care Cancer.

